# Effects of water surface conditions on standing wave fields generated inside a small chamber

**DOI:** 10.1186/2050-5736-2-S1-A23

**Published:** 2014-12-10

**Authors:** Nobuki Kudo

**Affiliations:** 1 Graduate School of Information Science and Technoligy, Hokkaido University, Japan

## 

In typical in vitro studies on sonoporation, cells were cultured in a Petri dish or a multi-well plate filled with culture medium and sonicated by an ultrasound transducer placed under the dish or well. In this condition, ultrasound wave is reflected at the surface of the culture medium, and a complex standing wave field is generated inside the dish. Precise dosimetry is essential in ultrasound therapy; however, it is difficult to explore the complex acoustic fields using a hydrophone. Especially, water height and water surface reflection that determine standing wave field is important. We have proposed a novel optical method visualizing ultrasound fields [1,2] In this study, the method was applied for visualization of standing wave fields inside a small chamber, and the effects of water surface reflection was investigated.

An optical system of focused shadowgraphy was used to take two shadowgrams with and without ultrasound exposure. Sensitive visualization was achieved by subtraction of these images. Burst ultrasound of 2 MHz in center frequency was irradiated from the bottom of a small cubic-shaped chamber that has the same cross-sectional shape as that of a Petri dish. In the first experiment, a water surface was covered with a plastic plate that enables ultrasound reflections at a right angle, and standing wave fields after five times reflection at the water surface and the bottom were visualized. Antinodes were visualized as bright lines at the interval of ?/2, and the maximum brightness was approximately twice of that visualized in the traveling wave condition, indicating the possibility for quantitative evaluation of the ultrasound pressure inside the chamber. Ultrasound fields were also visualized without the reflector at the water surface. In this condition, the surface was disturbed by a convection flow generated by radiation force of the ultrasound, and reflection of the ultrasound became unstable. Unstable changes in positions of nodes and antinodes were visualized, indicating water surface conditions have serious effects on the ultrasound fields inside Petri dish. This study was partially supported by JSPS KAKENHI, 23300182 and 23650247.

